# Multi-Concept Frailty Predicts the Late-Life Occurrence of Cognitive Decline or Dementia: An Updated Systematic Review and Meta-Analysis of Longitudinal Studies

**DOI:** 10.3389/fnagi.2022.855553

**Published:** 2022-05-11

**Authors:** Chun-Yan Guo, Zhen Sun, Chen-Chen Tan, Lan Tan, Wei Xu

**Affiliations:** Department of Neurology, Qingdao Municipal Hospital, Qingdao University, Qingdao, China

**Keywords:** dementia, cognitive decline, physical frailty, cognitive frailty, social frailty, biopsychosocial frailty, risk factor

## Abstract

**Background:**

Frailty is a multidimensional syndrome that increases an individual’s vulnerability for developing adverse health outcomes, which include dementia. It might serve as a promising target for dementia prevention. However, there are currently no studies summarizing the association between multi-concept frailty and the risk of cognitive disorders. This study aims to summarize the evidence of associations between multi-concept frailty and cognitive disorders based on longitudinal studies.

**Methods:**

Scopus, The Cochrane Library, PsycINFO, CINAHL, PubMed, and EMBASE databases were searched from inception to January 2, 2022. Longitudinal studies, which explored the association of frailty with incident risk of cognitive decline or dementia, were included. The multivariable-adjusted effect estimates were pooled by random-effects models. The evidence credibility was depicted according to the Grading of Recommendations Assessment, Development, and Evaluation (GRADE) method.

**Results:**

A total of 30 longitudinal studies were included. Four types of frailty concepts were involved, including physical, cognitive, social, and biopsychosocial frailty. The meta-analysis comprised 20 studies of 252,571 older adults (mean age: 64.1–80.4 years), among whom 7,388 participants developed cognitive decline or dementia. Physical frailty was associated with higher risk of developing cognitive disorders [pooled relative risk (pRR) = 1.52, 95% confidence interval (CI): 1.28–1.80, *I*^2^ = 21.2%, pRR = 1.62 for cognitive decline, 95% CI: 1.07–2.45, *I*^2^ = 40.2%, pRR = 1.37 for all-cause dementia (ACD), 95% CI: 1.13–1.66, *I*^2^ = 0.0%]. Cognitive frailty (pRR = 2.90, 95% CI: 1.28–6.55, *I*^2^ = 78.1%) and pre-frailty (pRR = 4.24, 95% CI: 2.74–6.56, *I*^2^ = 30.2%) were linked to higher risk of ACD. Biopsychosocial frailty could predict a 41% (pRR = 1.41, 95% CI: 1.17–1.71) elevated risk of cognitive decline or dementia [pRR = 1.53 (95% CI: 1.19–1.96) for ACD and 1.11 (95% CI: 1.05–1.17) for Alzheimer’s disease (AD)]. In the systematic review, social frailty was associated with a 53% higher risk of AD. Preventing frailty could avoid a maximum of 9.9% cognitive disorders globally. The overall evidence strength is rated as low-to-moderate. Inconsistency and imprecision are major sources of bias.

**Conclusion:**

Frailty in late life is a promising risk factor for cognitive disorders. Frail elderly should be monitored for their cognitive dynamics and initiate early prevention of dementia.

**Systematic Review Registration:**

www.ClinicalTrials.gov, identifier CRD4202127 3434.

## Introduction

Around 55 million people are living with dementia worldwide and there are nearly 10 million new cases every year. The impact of dementia on individuals, families, and society can be physical, psychological, social, and economic (WHO, 2022). Medications for treating dementia produce limited clinical benefits (Orrell and Brayne, 2015; Winblad et al., 2016), it is, thus, particularly important to identify potentially modifiable risk factors, which can help predict and/or prevent dementia. The etiology of dementia is multifactorial. A new life-course model reported the twelve potentially modifiable risk factors for dementia, which accounted for around 40% of worldwide dementias: less education, hypertension, hearing impairment, smoking, obesity, depression, physical inactivity, diabetes, low social contact, excessive alcohol consumption, traumatic brain injury, and air pollution (Livingston et al., 2020). At present, the multi-intervention strategy with multiple targets has been proposed to be the most promising way for Alzheimer’s disease (AD) prevention. Thus, we have reason to believe that an integrated indicator, a developing indicator that takes into account all risk factors for dementia, should have an optimal ability for predicting dementia.

Frailty is a multidimensional syndrome reflecting a non-specific state of vulnerability and a multisystem change (Morley et al., 2013). It is an integrated indicator and might serve as a promising target for dementia prevention. A cross-sectional clinicopathological study showed the degree of frailty among people of the same age modified the association between AD pathology and AD, since individuals with even a low level of AD pathology might be at risk for dementia if they had high amounts of frailty (Wallace et al., 2019). Recently, another cross-sectional clinicopathological study suggested that frailty was associated with dementia status independently of neuropathological burden. Preventing severe frailty could avoid 14.2% of dementia cases (Wallace et al., 2021). Besides, the result of a randomized clinical trial confirmed that physical exercise can reverse frailty and improve cognitive function (Tarazona-Santabalbina et al., 2016). In the last decades, although more than forty operational definitions have been proposed about frailty, these can be summarized in four major conceptual models according to constituent elements: physical frailty, cognitive frailty, social frailty, and biopsychosocial frailty. Physical frailty is a medical syndrome that is characterized by diminished strength, endurance, and reduced physiologic function (Morley et al., 2013). Some evidence showed that physical frailty may be closely associated with cognitive impairment (Panza et al., 2015), and one person would be judged to be cognitively frailty if he has both physical frailty and cognitive impairment without dementia (Kelaiditi et al., 2013). Social frailty is a continuum of being at risk of losing, or having lost, social and general resources, activities, or abilities that are important for fulfilling basic social needs (Bunt et al., 2017). Biopsychosocial frailty considers the integral functioning of individuals, and it is a broader concept that covers frailty factors in physical, social, and psychological dimensions (Gobbens et al., 2010; de Vries et al., 2011; Panza et al., 2019).

Though longitudinal studies explored associations between varying concepts of frailty and cognitive disorders [cognitive decline, all-cause dementia (ACD), or AD], the conclusion is largely debated. The present study aims to meta-analyze the relationships of frailty with the risk of developing cognitive disorders based on evaluating the evidence’s credibility.

## Methods

### Search Strategy and Selection Criteria

We followed the recommendations by the Preferred Reporting Items for Systematic Reviews and Meta-Analyses 2009 guidelines (Stroup et al., 2000; Moher et al., 2010). Scopus, The Cochrane Library, PsycINFO, CINAHL, PubMed, and EMBASE were searched until January 2, 2022 (final update) using the strategy: (longitudinal OR cohort OR prospective OR retrospective OR nested case-control) AND (cognitive OR dementia OR Alzheimer OR cognition), AND (frailty OR frail). Bibliographies of relevant original studies and systematic reviews were hand-searched in case of omission. The inclusion criteria were as follows: (a) Study was designed as a population-based longitudinal study; (b) participants were adults without dementia at baseline; (c) frailty status was examined at baseline; and (d) studies reported the association of frailty status with risk of developing dementia or cognitive decline. Exclusion criteria includes: (a) Reviews or conference abstracts; (b) cross-sectional studies; and (c) postoperative cognitive dysfunction. We did not restrict the language category when searching for literature. If studies were based on an identical population, the study with a larger sample size was included. Literature selection was performed by two experienced investigators (Guo CY and Xu W) and any disagreements were resolved by consensus and arbitration within the review team.

### Data Extraction

Predesigned templates were used to extract the data, including general items (first author, publication year, and country), study design (prospective/retrospective cohort or nested case-control study), sample source (community organization, or others), participation rate at baseline (generalizability), mean age, female percentage, baseline cognitive status (free of dementia, mild cognitive impairment, or cognitively intact), sample size and incident case number for analysis, frailty type and assessment approach, outcome and diagnostic criteria, follow-up duration, attrition rate, adjusted confounders, and the multivariable-adjusted risk estimates. The data extraction was performed by two experienced investigators (Guo CY and Xu W) and any discrepancies were addressed by negotiation within the review team.

### Assessment of the Study Quality and Credibility of Meta-Analyses

An evolving Newcastle-Ottawa Quality Assessment Scale (NOS) for observational cohort studies was employed to evaluate the quality of eligible studies (Yu et al., 2020). The score for each item evaluated the risk of bias in sample selection, confounding bias, and outcome (Supplementary Appendix 1). Quality evaluation was performed by two investigators (Guo CY and Xu W) and any disagreements were resolved by consensus and arbitration within the team. The total score of NOS was regarded here as a proxy to assess the overall risk of bias for every single study. The credibility of meta-analyses was appraised according to the Grading of Recommendations Assessment, Development, and Evaluation (GRADE) criteria of inconsistency, imprecision, risk of bias, publication bias, and indirectness (Gopalakrishna et al., 2014). Inconsistency refers to heterogeneity. Imprecision refers to random error. The risk of bias was evaluated by a weighted NOS score. The source of indirectness, herein, is the use of surrogate endpoints in place of the outcome—dementia (Supplementary Appendix 2).

### Statistical Analyses

When both the multivariable-adjusted model and the model without adjusted confounding factors were included in one study, we selected the effect estimates of the former model (Supplementary Table 1). Multivariable-adjusted OR, RR, or HR with 95% CI of risks of cognitive disorders for frailty compared with non-frailty were extracted from the included studies. Risk estimates and 95% CI were logarithmically transformed and pooled using random models (DerSimonianLaird method) (Higgins et al., 2003). We use the following formula to convert ORs to RRs because ORs is inclined to overvalue the effect’s sizes compared with RRs/HRs (Grant, 2014).


$$R\,R_{a\,d\,j\,u\,s\,t\,e\,d}=\frac{O\,R_{a\,d\,j\,u\,s\,t\,e\,d}}{\left(1-P_{0}\right)+\left(P_{0}\,t\,i\,m\,e\,s\,O\,R_{a\,d\,j\,u\,s\,t\,e\,d}\right)}$$

P_0_ is the incidence of the outcome in the non-frail group, and the incidence rate of the total sample would be used if P_0_ was not accessible (Grant, 2014). We calculated a 95% prediction interval to assess the precision of the result (Riley et al., 2011). The heterogeneity across the studies was assessed by chi-square test, and considered as present if the *P-*value was less than 0.1. Heterogeneity was classified as possibly low (0–30%), moderate (30–60%), and substantial (60–100%) in the present study. The degree of heterogeneity was analyzed using the I-square (*I*^2^) statistic. If the number of publications included in the meta-analysis is greater than or equal to ten, the source of heterogeneity will be explored *via* sensitivity analyses and subgroup analyses according to multiple variables, including sample source, study design, sample size, cognitive status at baseline, and quality score. We also used meta-regression to explore the influence of the follow-up period and the diagnostic method of outcomes on effect size. Egger’s test was carried out to assess publication bias. Finally, population attributable risk (PAR) was calculated using the method by Barnes and Yaffe (2011) to estimate the percentage of total cognitive disorders attributable to frailty in the global population. Meta-analysis was conducted using the Stata 12.0 for windows (StataCorp LP, College Station, Texas, United States).

## Results

### Searching Results and Characteristics of Studies

**FIGURE 1:**
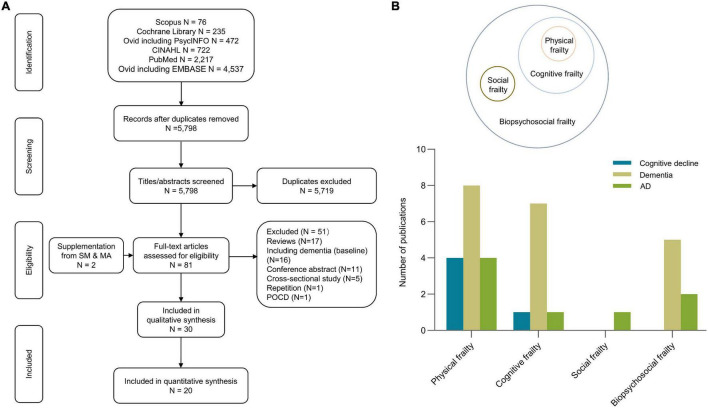
Search flowchart **(A)** and summary characteristics of included studies **(B)**. The search yielded 5,798 literatures after deduplication. After scanning the titles and abstracts, 79 articles were considered as potentially eligible. After reviewing the bibliography and full-texts, 30 studies met the eligibility criteria, and 20 studies reporting risk estimates were included in the meta-analyze. AD, Alzheimer’s disease; POCD, postoperative cognitive dysfunctive; SM and MA, systematic review and meta-analysis.

Figure 1A exhibits the flow diagrams of the study selection process. The search yielded 5,798 articles after deduplication. After scanning the titles and abstracts, 79 articles were considered as potentially eligible. After reviewing the bibliography and full-texts, 30 studies met the eligibility criteria, and 20 studies reporting risk estimates were included in the meta-analysis (Buchman et al., 2007; Avila-Funes et al., 2009, 2012; Boyle et al., 2010; Gray et al., 2013; Solfrizzi et al., 2013, 2017a,2017b,^2019^; Montero-Odasso et al., 2016; Feng et al., 2017; Rogers et al., 2017; Trebbastoni et al., 2017; Chen et al., 2018; Shimada et al., 2018a,b; Li et al., 2020; Sugimoto et al., 2020; Bai et al., 2021; Ward et al., 2021) cognitive domains (Boyle et al., 2010; Bunce et al., 2019; Magnuson et al., 2019; Thibeau et al., 2019; Gale et al., 2020; Paolillo et al., 2020; Chen et al., 2021; Williams et al., 2021), two literature investigated the association between social frailty and cognition (Tsutsumimoto et al., 2019; Huang et al., 2021), and one literature investigated the correlation between cognition and frailty status transitions (Liu et al., 2021). The detailed characteristics of studies included in the systematic review and meta-analysis are summarized in Table 1 (for more details see Supplementary Table 1). Studies included in the meta-analysis reported three types of frailty concepts, including physical, cognitive, and biopsychosocial frailty (Figure 1B). The corresponding assessment scale of diverse frailty is presented in Supplementary Table 2. Twelve (60%) studies analyzed the effect of physical frailty on cognitive decline (Boyle et al., 2010; Montero-Odasso et al., 2016; Feng et al., 2017; Chen et al., 2018) (20%), ACD (Avila-Funes et al., 2012; Gray et al., 2013; Solfrizzi et al., 2013, 2017a; Montero-Odasso et al., 2016; Shimada et al., 2018a,b; Li et al., 2020) (40%), or AD (Buchman et al., 2007; Avila-Funes et al., 2012; Gray et al., 2013; Solfrizzi et al., 2013) (20%). Seven (35%) studies investigated the relationship between cognitive frailty and ACD (Avila-Funes et al., 2009; Montero-Odasso et al., 2016; Solfrizzi et al., 2017a,b; Shimada et al., 2018a,b; Sugimoto et al., 2020) (35%), AD (Solfrizzi et al., 2017b) (5%), or cognitive decline (Montero-Odasso et al., 2016) (5%). Six (30%) studies reported the connection between biopsychosocial frailty and ACD (Rogers et al., 2017; Solfrizzi et al., 2019; Li et al., 2020; Bai et al., 2021; Ward et al., 2021) (25%) or AD (Trebbastoni et al., 2017; Solfrizzi et al., 2019) (10%).

**TABLE 1:** Characteristics of included studies.

References	Country	Sample; case	Mean age; female	Cognitive status at baseline	Type of frailty	Frailty assessment	Interesting outcome and its diagnostic criteria	Follow-up	NOS
Buchman et al. (2007)	Chicago	823; 89	80.4; 74.6%	Free of dementia	PF	mFP	**AD:** NINCDS-ADRDA	3 y (mean)	7
Avila-Funes et al. (2009)	France	4,827; 157	74.1*; 61.2%*	Free of dementia	PF; CF	mFP; PF+CI (subjects in the lowest quartile in MMSE and IST)	**Dementia:** DSM-IV	4 y (max)	8
Boyle et al. (2010)	Chicago	761; 305	79; 76%	Cognitively normal	PF	A score based on grip strength, timed walk, body composition and fatigue	**MCI:** CI and without dementia (NINCDS-ADRDA); Performance in cognitive domains.	12 y (max)	6.5
Avila-Funes et al. (2012)	France	5,480; 388	74; 61.7%	Free of dementia	PF	mFP	**Dementia:** DSM-IV; **AD:** NINCDS-ADRDA	7 y (max)	8
Gray et al. (2013)	United States	2,619; 521	76.8; 60.1%	Free of dementia	PF	mFP	**Dementia:** DSM-IV; **AD:** NINCDS-ADRDA	6.5 y (mean)	7.5
Solfrizzi et al. (2013)	Italy	2,581; 65	73.07; 45.18%	Cognitively normal	PF	mFP	**Dementia:** DSM-III; **AD:** NINCDS-ADRDA	3.9 y (median)	8
Montero-Odasso et al. (2016)	Canada	252; 53	76.7; 62.7%	Free of dementia	PF; CF	mFP; PF+CI (MoCA<26 and CDR = 0.5)	**Cognitive decline:** at least 2 points decrease of MoCA score; **Dementia:** DSM-IV and CDR ≥ 1	1.5 y (mean); 5 y (max)	6
Feng et al. (2017)	Singapore	1,491; 105	66*; 64.8%*	Cognitively normal	PF	mFP	**Cognitive decline:** MMSE ≤ 23;	3 y (max)	7
Rogers et al. (2017)	United Kingdom	8,722; 365	64.4; 54.9%	Free of dementia	BF	Multidimensional FI (>0.25)	**Dementia:** Self-report	9.4 y (mean)	7
Solfrizzi et al. (2017a)	Italy	2,373; 43	72.8; 44.5%	Free of dementia	PF; Potentially reversible CF	mFP; PF +MCI	**Dementia:** DSM-III	3.5 y (max)	7
Solfrizzi et al. (2017b)	Italy	2,150; 171	73.2; 42.89%	Free of dementia	Reversible CF	PF (mFP) +SCD (MMSE ≥ 15 + impairs on GDS-30 item 14)	**Dementia:** DSM-III; **AD:** NINCDS-ADRDA	7 y (max)	8
Trebbastoni et al. (2017)	Italy	91; 58	72.7; 49.47%	MCI	BF	A score based on multidimensional FI	**AD:** NIA-AA	5 y (max)	5
Chen et al. (2018)	Japan	708; 159	72.6; 40.3%	Free of dementia	PF	mFP	**Cognitive decline:** at least two points decrease of MoCA score	2 y (max)	7
Shimada et al. (2018a)	Japan	4,570; 241	71.6; 51.51%	Free of dementia	PF; CF	Slowness or muscle weakness; PF+CI (deficits on ≥ 1 NCGG-FAT’s domains)	**Dementia:** ICD-10	3 y (max)	7.5
Shimada et al. (2018b)	Japan	4,072; 81	71.59; 51.58%	Free of dementia	PF; CF	mFP; PF+CI (deficits on ≥ 2 NCGG-FAT’s domains)	**Dementia:** ICD-10	2 y (max)	7.5
Bunce et al. (2019)	Australia	896; …	na; 49.11%	Free of dementia	PF	mFP	Performance in specific cognitive domains	12 y (max)	7
Magnuson et al. (2019)	United States	610; …	59.36; 100%	Cognitively normal	PF	A modified score based on mFP	Performance in specific cognitive domains	7 m (max).	5
Solfrizzi et al. (2019)	Italy	2,171; 182	73.3; 43.13%	Cognitively normal	BF	PF (mFP) +impairs on ≥ 1 items of GDS-30 3 or 10	**Dementia:** DSM-III; **AD:** NINCDS-ADRDA	7 y (max)	8.5
Thibeau et al. (2019)	Canada	632; …	70.7; 66.7%	Free of dementia	PF	A score based on physical FI	Performance in specific cognitive domains	Na	7
Tsutsumimoto et al. (2019)	Japan	3,720; 192	71.7; 51.56%	Cognitively normal	Social frailty	Frailty: with ≥ 2 components^#^	**AD:** ICD-10	53 m (max); 51.5 m (mean)	7.5
Gale et al. (2020)	United Kingdom	950; …	70; 50.74%	Cognitively normal	PF	mFP	Performance in specific cognitive domains	9 y (max)	6.5
Li et al. (2020)	China	2,022; 206	72.8; 55.89%	Free of dementia	PF; BF	mFP or physical FI ≥ 0.25; Multidimensional FI ≥ 0.25	**Dementia:** The 10/66 dementia diagnosis	5 y (mean)	8
Paolillo et al. (2020)	United States	110; …	51.08; 21.82%	Cognitively normal	PF	mFP	Performance in specific cognitive domains	2 y (max)	6.5
Sugimoto et al. (2020)	Japan	248; 82	76.3; 60.89%	MCI	Potentially reversible CF	PF (physical FI ≥ 0.25) +MCI (NIA-AA)	**Dementia:** NIA-AA	3 y (max); 2.5 y (median)	6
Williams et al. (2021)	United States	845; …	29.69; 47.93%	Free of dementia	PF	mFP	Performance in specific cognitive domains	5 y (max)	6
Bai et al. (2021)	Sweden	10,487; 2,355	72.3; 56.00%	Free of dementia	BF	A score based on multidimensional FI	**Dementia:** DSM-III-R and DSM-IV	19 y (max)	7.5
Chen et al. (2021)	Taiwan	521; …	72.7; 52.4%	Free of dementia	PF; Psychosocial frailty	mFP; Frailty: integrating self-rated health, mood, social contact	Performance in specific cognitive domains	4 y (max)	6.5
Liu et al. (2021)	China	196; …	83.7; 57.8%	Free of dementia	PF	FRAIL Scale	The correlation between IC domains and frailty	2 y (max)	6.5
Ward et al. (2021)	Canada	196,123; 1,762	64.1; 53%	Free of dementia	BF	A score based on multidimensional FI	**Dementia:** ICD-9 and ICD-10	8 y (median)	8
Huang et al. (2021)	Japan	663; …	69.5; 56.7%	Free of dementia	Social frailty	Frailty: with ≥ 2 components^&^	The association between social frailty and IC	3 y (max)	6.5

*As the information of sample wasn’t accessible, the total participation information was used as a proxy.

*^#^The components included going out less, not visiting friends, not feeling helpful to others, living alone, and not talking every day.*

*^&^The components included financial difficulty, living alone, non-participation in social activities, not regular contacting with others.*

*AD, Alzheimer’s disease; BF, biopsychosocial frailty; CDR, Clinical Dementia Rating; CF, cognitive frailty; CI, Cognitive impairment; DSM, Diagnostic and Statistical Manual of Mental Disorders; FI, frailty index; FRAIL, fatigue, resistance, ambulation, illnesses, and loss of weight; GDS, Geriatric Depression Scale; HIV, human immunodeficiency virus; IC, intrinsic capacity; ICD-10, International Classification of Diseases-10; IST, Isaacs Set Test; MCI, mild cognitive impairment; mFP, modified frailty phenotype; MMSE, Mini-Mental State Examination; MoCA, Montreal Cognitive Assessment; m, month; na, not applicable; NCGG-FAT, National Center for Geriatrics and Gerontology Functional Assessment Tool; NIA-AA, National Institute on Aging-Alzheimer’s Association criteria; NINCDS-ADRDA, National Institute of Neurological and Communicative Disorders and Stroke and the Alzheimer’s Disease and Related Disorders Association; PF, physical frailty; SCD, subjective cognitive decline; y, year.*

### Physical Frailty and Cognitive Disorders

**FIGURE 2:**
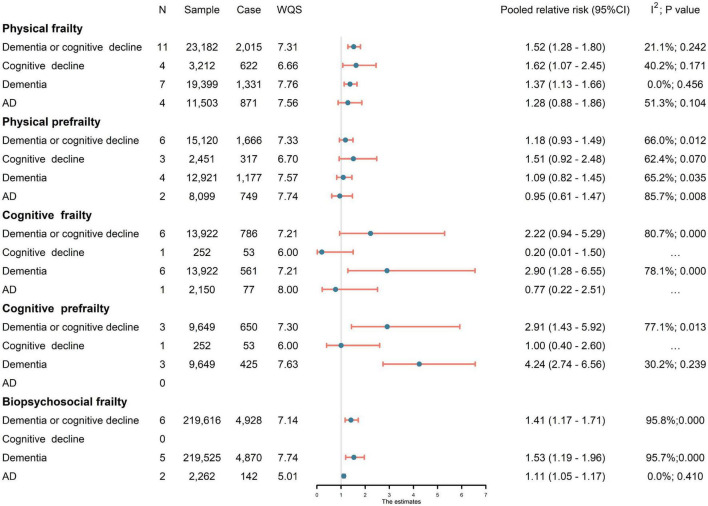
Association of frailty with risk of cognitive disorders. Since physical frailty involved both prefrailty (a condition between frail and non-frail) and frailty (Morley et al., 2013), cognitive frailty involved both cognitive prefrailty and cognitive frailty. AD, Alzheimer’s disease; CI, confidence interval; N, number of studies; WQS, weighted quality score.

Meta-analysis of eleven studies (23,182 subjects) showed that physical frailty was significantly associated with an increased risk of developing cognitive disorders (pRR = 1.52, 95% CI: 1.28–1.80, *I*^2^ = 21.1%). The relationship remained significant for ACD (pRR = 1.37, 95% CI: 1.13–1.66, *I*^2^ = 0.0%) or cognitive decline (pRR = 1.62, 95% CI: 1.07–2.45, *I*^2^ = 40.2%), while no association was revealed for AD (pRR = 1.28, 95% CI: 0.88–1.86, *I*^2^ = 51.3%). No significant association was revealed between physical prefrailty and cognitive disorders (Figure 2). Sensitivity analysis, by excluding one study each time, barely changed the primary result. No subgroup difference was revealed for sample source, study design, sample size, baseline cognitive status, or quality score (Supplementary Figure 1). Meta-regression revealed that the follow-up period and the diagnostic method of outcomes had no significant influence on effect size.

### Cognitive Frailty and Cognitive Disorders

Six studies (13,922 subjects) were pooled in analyses for the effects of cognitive frailty. Cognitive frailty could predict significantly higher risk of incident of ACD (pRR = 2.90, 95% CI: 1.28–6.55, *I*^2^ = 78.1%). Moreover, meta-analysis of three studies (9,649 subjects) revealed that cognitive pre-frailty was also associated with higher risk of cognitive disorders (pRR = 2.91, 95% CI:1.43–5.92, *I*^2^ = 77.1%). The risk estimate of cognitive pre-frailty people was especially large for ACD (pRR = 4.24, 95% CI: 2.74–6.56, *I*^2^ = 30.2%).

### Biopsychosocial Frailty and Cognitive Disorders

Six studies (219,616 subjects) investigated the impact of biopsychosocial frailty. Biopsychosocial frailty had significant effect on cognitive disorders (pRR = 1.41, 95% CI: 1.17–1.71; *I*^2^ = 95.8%), the larger effect was found on dementia (pRR = 1.53, 95% CI: 1.19–1.96; *I*^2^ = 95.7%), and biopsychosocial frailty also contributed to a 11% higher risk of AD (pRR = 1.11, 95% CI: 1.05–1.17; *I*^2^ = 0.0%).

### Systematic Review

As shown in Supplementary Table 3, eight studies explored relationships between frailty and performance in specific cognitive domains over time. In old people (mean age: 59.4–79 years), physical frailty was associated with a more rapid decline in memory and visuospatial ability, but physical frailty did not affect verbal fluency. There wasn’t a consistent conclusion on the relation of physical frailty to speed or executive function. Two studies of social frailty have shown that social frailty was connected with a 53% higher risk of AD (Tsutsumimoto et al., 2019) and cognitive decline was greater in the social pre-frailty or frailty group than robustness group (Huang et al., 2021). In addition, a new study indicated (Liu et al., 2021) that cognitive impairment was connected to the transitions from non-frail to physical frail status.

### Credibility of Meta-Analyses

**FIGURE 3:**
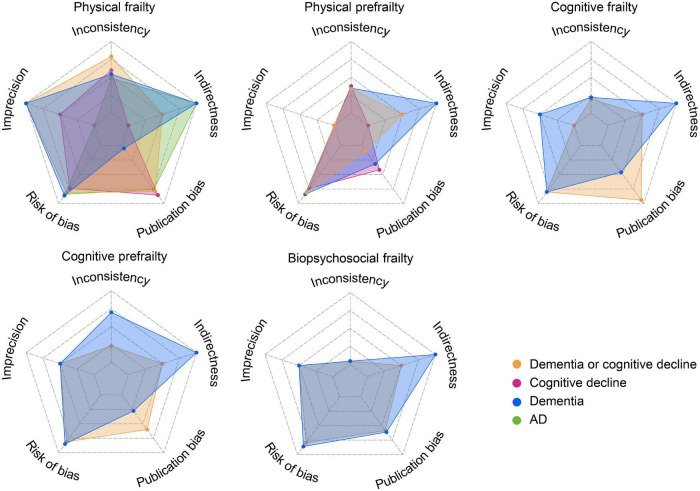
Credibility of meta-analyses results (for more details see Supplementary Table 4). The credibility improved with the increased area of the radar map.

In general, the evidence robustness is low-to-moderate. Heterogeneity was obvious in meta-analyses about physical prefrailty, cognitive frailty, and biopsychosocial frailty. Imprecision is a common problem for analyses of physical prefrailty, cognitive frailty, cognitive prefrailty, and biopsychosocial frailty. A small number of publications, a diverse approach of frailty assessment, limited generalizability, follow-up inadequacy, and attrition are major sources of bias (Figure 3).

### Population Attributable Risk

We computed PAR for three types of frailty for which global prevalence was accessible as follows: physical frailty (12%) (O’Caoimh et al., 2021), cognitive prefrailty/frailty (9%) (Qiu et al., 2022), and biopsychosocial frailty (26.8%) (Veronese et al., 2021). The PAR was 5.87, 4.22, and 9.90%, respectively.

## Discussion

The present study indicated that non-demented elderly with frailty (including physical, cognitive, social, and biopsychosocial frailty) were at higher risk of developing dementia, though the evidence strength is limited by inconsistency and imprecision. Compared to previous publications (Borges et al., 2019; Bu et al., 2021; Chu et al., 2021), the present study had several advantages: (1) The topic is comprehensive covering all aspects of frailty, including the “biopsychosocial model”; (2) more evidence was incorporated (10 more longitudinal studies); (3) the results were translated based on evidence evaluation, we evaluated the robustness of the evidence for each association and provided clues for further research in this field.

Physical frailty is a condition in which the individual experiences losses in the physical domains of human functioning. We found physical frailty is an important risk factor for cognitive disorders. The underlying mechanisms might be explained by common risk factors shared between physical frailty and cognitive decline. Specifically, common risk factors included brain neuropathology (neurofibrillary tangles, β-amyloid load, nigral neuronal loss, genetic mutations, and cerebral atrophy), hormonal dysregulation (reduced testosterone and insulin resistance), cardiovascular risk (diabetes, dyslipidemia, and hypertension), psychological and environmental factors (depression and nutritional deficiencies) and chronic inflammation (Kelaiditi et al., 2013; Robertson et al., 2013; Gallucci et al., 2018). In studies investigating correlations between frailty and performance in specific cognitive domains in the elderly, physical frailty was associated with memory decline. It is possible that neuropathological effects produced by the above risk factors of physical frailty could affect the hippocampus [associated with memory (Panegyres, 2004)] and result in cognitive disorders. Alterations in hippocampal synaptic function, neuronal membrane properties, and axonal trajectories, which might lead to dementia, have been reported for people with frailty (Bishop et al., 2010). Little work has directly explored mechanisms underlying this link, so experimental evidence is needed to support these possible mediators. Liu and colleagues found that impairment in cognition was associated with the transitions from non-frail to physical frail status (Liu et al., 2021), suggesting that the relationship between physical frailty and cognitive ability may be bi-directional.

Cognitive frailty is a state characterized by cognitive impairment due to physical conditions not the presence of concomitant neurological disease (Kelaiditi et al., 2013). According to our study, cognitive frailty had a stronger predictive validity on dementia than physical frailty. To determine the primary and secondary preventative measures for cognitive frailty, Ruan proposed two subtypes of cognitive frailty based on the severity of cognitive impairment: the reversible and the potentially reversible (the definitions are presented in Box 1; Ruan et al., 2015). Solfrizzi found that the reversible cognitive frailty was a short- and long-term predictor of overall dementia (Solfrizzi et al., 2017b), while no association was revealed between the potentially reversible cognitive frailty and dementia (Solfrizzi et al., 2017a). Our results indicated that cognitive prefrailty is also a vital predictor of cognitive disorders. It may be a useful indicator for identifying early signs of dementia and a promising intervention target for dementia prevention.

**Box 1. Definitions of multi-concept frailty.**

• Physical frailty/prefrailty is a medical syndrome characterized by diminished strength, endurance, and reduced physiologic function that increases an individual’s vulnerability for adverse health-related outcomes.

• Cognitive frailty/prefrailty is a state characterized by cognitive impairment due to physical conditions not the presence of concomitant neurological disease.

• Potentially reversible cognitive frailty is a clinical syndrome of mild cognitive impairment caused by physical factors (e.g., physical frailty).

• Reversible cognitive frailty is a clinical syndrome of subjective cognitive decline caused by physical factors.

• Social frailty is a continuum of being at risk of losing, or having lost, social and general resources, activities, or abilities that are important for fulfilling basic social needs.

• Biopsychosocial frailty is a broader concept that covers frailty factors in physical, social, and psychological dimensions.

Social frailty can be defined as the absence of social resources, social activities, and self-management abilities (Bunt et al., 2017). There are several potential mechanisms between social frailty and cognition at present. First, social stress may affect hormones, immune functioning, and inflammatory processes and induce cognitive decline, social interactions and social support may buffer physiological reactions to stress (Fratiglioni et al., 2000; Cohen, 2004; Agrigoroaei and Lachman, 2011). Second, rich social networks contribute to cognitive function exercise and provide easier access to health information (Berkman et al., 2000; Tsutsumimoto et al., 2019). Third, social frailty was independently associated with poor physical function (Tsutsumimoto et al., 2017), we presume it could lead to cognitive decline through physical frailty.

The three dimensions of frailty (physical, social and psychological) are interrelated rather than independent (Teo et al., 2019), and biopsychosocial frailty fully reflected the multidimensional nature of frailty. However, the risk estimates of cognitive disorders in biopsychosocial frailty individuals were smaller than physical frailty or cognitive frailty in our results. Firstly, follow-up periods of the included studies for biopsychosocial frailty ranged from 5 to 8.5 years and maybe too short to capture overall predictive properties of frailty. Biopsychosocial frailty was evaluated by multidimensional frailty index (FI) in 5 included articles and a previous study showed that frailty based on FI better-predicted dementia over 10 years rather than over 5 years. Items related to health defects for developing multidimensional FI may be partial, leading to smaller risk estimates. Our results showed that multidomain interventions might take an important part in delaying the future appearance of cognitive disorders and secondary occurrence of adverse health-related outcomes, such as disability, hospitalization, and mortality. There have been trials showing that physical exercise can treat frailty and improve cognitive function. The lifestyle interventions and independence for elders study (LIFE)-randomized clinical trial, involving 1,298 participants with cognitive frailty, suggested that physical activity reduced the severity of cognitive frailty compared with a health education program (Liu et al., 2018). Another randomized multicenter control trial proved that physical exercise had benefits for cognitive status among physical pre-frail/frail patients with mild cognitive impairment or dementia (Casas-Herrero et al., 2019). Furthermore, the findings from another randomized trial ascertained a supervised-facility multicomponent exercise program can reverse physical frailty and improve cognitive function (Tarazona-Santabalbina et al., 2016). Physical exercise should be regarded as a vital component of multidomain interventions. Better results may be produced if physical exercise is combined with interventions in other areas.

Our results suggested a frail state may indicate the onset of cognitive decline; however, the results are subject to some limitations in practical application. First, heterogeneity of frailty diagnostic criteria and diagnostic methods of the outcome. Diverse diagnostic criteria of frailty had been used now. Physical frailty is diagnosed as physical FI ≥ 0.25 or conforms to the standard of frailty phenotype, while biopsychosocial frailty is evaluated based on multidimensional FI in most cases. Nevertheless, the items included in FI and diagnostic criteria of social and cognitive frailty are ambiguous. Second, the threshold for assessing frailty or non-frailty varied from study to study, which could significantly affect the proportions classified as frail or not. However, we cannot investigate thresholds and potential impacts because the studies used diverse frailty scales and evaluation criteria. Third, participants from organizations such as medical centers tended to have more risk factors of cognitive decline than community residents. That may decrease the representation of the total population. Fourth, the length of follow-up included in meta-analysis ranged from 2 to 15 years, insufficient follow-up time in some studies may reduce risk estimates of cognitive disorders.

Based on the credibility of our meta-analyses, we propose several suggestions for future research. Firstly, developing authoritative screening scales with higher sensitivity and specificity for cognitive frailty and biopsychosocial frailty, and controlling confounding factors may sufficiently help reduce inconsistency. Furthermore, expanding sample size or random sampling from community residents could promote the generalizability of the conclusion. Frailty and cognitive changes should be measured simultaneously in longitudinal studies with adequate follow-up to test for reverse causation or lead-lag effects.

In conclusion, the present study suggested that frailty is significant to help identify populations at high risk of cognitive disorders. Frail elderly should be regarded as the primary target of resource allocation in the prevention and treatment of dementia.

## Data Availability Statement

The original contributions presented in the study are included in the article/Supplementary Material, further inquiries can be directed to the corresponding author/s.

## Author Contributions

WX: conceptualization and design of the study, and revision of the manuscript. C-YG: collection and analysis of the data, drafting and revision of the manuscript, and preparation of all the figures. C-CT: collection of the data. ZS and LT: revision of the manuscript. All authors contributed to the article and approved the submitted version.

## Conflict of Interest

The authors declare that the research was conducted in the absence of any commercial or financial relationships that could be construed as a potential conflict of interest.

## Publisher’s Note

All claims expressed in this article are solely those of the authors and do not necessarily represent those of their affiliated organizations, or those of the publisher, the editors and the reviewers. Any product that may be evaluated in this article, or claim that may be made by its manufacturer, is not guaranteed or endorsed by the publisher.

## References

[B1] Agrigoroaei S. Lachman M. E. (2011). Cognitive functioning in midlife and old age: combined effects of psychosocial and behavioral factors. *J. Gerontol. B Psychol. Sci. Soc. Sci.* 66(Suppl. 1) i130–i140. 10.1093/geronb/gbr017 21743046 PMC3132767

[B2] Avila-Funes J. A. Amieva H. Barberger-Gateau P. Le Goff M. Raoux N. Ritchie K. (2009). Cognitive impairment improves the predictive validity of the phenotype of frailty for adverse health outcomes: the three-city study. *J. Am. Geriatr. Soc.* 57 453–461. 10.1111/j.1532-5415.2008.02136.x 19245415

[B3] Avila-Funes J. A. Carcaillon L. Helmer C. Carriere I. Ritchie K. Rouaud O. (2012). Is frailty a prodromal stage of vascular dementia? Results from the three-city study. *J. Am. Geriatr. Soc.* 60 1708–1712. 10.1111/j.1532-5415.2012.04142.x 22985143

[B4] Bai G. Wang Y. Kuja-Halkola R. Li X. Tomata Y. Karlsson I. K. (2021). Frailty and the risk of dementia: is the association explained by shared environmental and genetic factors? *BMC Med.* 19:248. 10.1186/s12916-021-02104-3 34657626 PMC8522144

[B5] Barnes D. E. Yaffe K. (2011). The projected effect of risk factor reduction on Alzheimer’s disease prevalence. *Lancet Neurol.* 10 819–828. 10.1016/s1474-4422(11)70072-2 21775213 PMC3647614

[B6] Berkman L. F. Glass T. Brissette I. Seeman T. E. (2000). From social integration to health: Durkheim in the new millennium. *Soc. Sci. Med.* 51 843–857. 10.1016/s0277-9536(00)00065-4 10972429

[B7] Bishop N. A. Lu T. Yankner B. A. (2010). Neural mechanisms of ageing and cognitive decline. *Nature* 464 529–535. 10.1038/nature08983 20336135 PMC2927852

[B8] Borges M. K. Canevelli M. Cesari M. Aprahamian I. (2019). Frailty as a predictor of cognitive disorders: a systematic review and meta-analysis. *Front. Med.* 6:26. 10.3389/fmed.2019.00026 PMC6389599 30838210

[B9] Boyle P. A. Buchman A. S. Wilson R. S. Leurgans S. E. Bennett D. A. (2010). Physical frailty is associated with incident mild cognitive impairment in community-based older persons. *J. Am. Geriatr. Soc.* 58 248–255. 10.1111/j.1532-5415.2009.02671.x 20070417 PMC3150526

[B10] Bu Z. Huang A. Xue M. Li Q. Bai Y. Xu G. (2021). Cognitive frailty as a predictor of adverse outcomes among older adults: a systematic review and meta-analysis. *Brain Behav.* 11:e01926. 10.1002/brb3.1926 PMC7821586 33159430

[B11] Buchman A. S. Boyle P. A. Wilson R. S. Tang Y. Bennett D. A. (2007). Frailty is associated with incident Alzheimer’s disease and cognitive decline in the elderly. *Psychosom. Med.* 69 483–489. 10.1097/psy.0b013e318068de1d 17556640

[B12] Bunce D. Batterham P. J. Mackinnon A. J. (2019). Long-term associations between physical frailty and performance in specific cognitive domains. *J. Gerontol.* 74 919–926. 10.1093/geronb/gbx177 29401240

[B13] Bunt S. Steverink N. Olthof J. van der Schans C. P. Hobbelen J. S. M. (2017). Social frailty in older adults: a scoping review. *Eur. J. Ageing* 14 323–334. 10.1007/s10433-017-0414-7 28936141 PMC5587459

[B14] Casas-Herrero A. Anton-Rodrigo I. Zambom-Ferraresi F. Sáez de Asteasu M. L. Martinez-Velilla N. Elexpuru-Estomba J. (2019). Effect of a multicomponent exercise programme (VIVIFRAIL) on functional capacity in frail community elders with cognitive decline: study protocol for a randomized multicentre control trial. *Trials* 20:362. 10.1186/s13063-019-3426-0 31208471 PMC6580555

[B15] Chen J. H. Shih H. S. Tu J. Chiou J. M. Chang S. H. Hsu W. L. (2021). A longitudinal study on the association of interrelated factors among frailty dimensions, cognitive domains, cognitive frailty, and all-cause mortality. *J. Alzheimers Dis.* 84 1795–1809. 10.3233/jad-215111 34719497

[B16] ChenS. HondaT. NarazakiK. ChenT. KishimotoH. HaeuchiY. (2018). Physical frailty is associated with longitudinal decline in global cognitive function in non-demented older adults: a prospective study. *J. Nutr. Health Aging* 22 82–88. 10.1007/s12603-017-0924-1 29300426

[B17] Chu W. Chang S. F. Ho H. Y. (2021). Adverse health effects of frailty: systematic review and meta-analysis of middle-aged and older adults with implications for evidence-based practice. *Worldviews Evid. Based Nurs.* 18 282–289. 10.1111/wvn.12508 34075676

[B18] Cohen S. (2004). Social relationships and health. *Am. Psychol.* 59 676–684. 10.1037/0003-066x.59.8.676 15554821

[B19] de Vries N. M. Staal J. B. van Ravensberg C. D. Hobbelen J. S. Olde Rikkert M. G. Nijhuis-van der Sanden M. W. (2011). Outcome instruments to measure frailty: a systematic review. *Ageing Res. Rev.* 10 104–114. 10.1016/j.arr.2010.09.001 20850567

[B20] Feng L. Nyunt M. S. Gao Q. Feng L. Lee T. S. Tsoi T. (2017). Physical frailty, cognitive impairment, and the risk of neurocognitive disorder in the Singapore longitudinal ageing studies. *J. Gerontol. A Biol. Sci. Med. Sci.* 72 369–375. 10.1093/gerona/glw050 27013397

[B21] Fratiglioni L. Wang H. X. Ericsson K. Maytan M. Winblad B. (2000). Influence of social network on occurrence of dementia: a community-based longitudinal study. *Lancet* 355 1315–1319. 10.1016/s0140-6736(00)02113-9 10776744

[B22] Gale C. Ritchie S. J. Starr J. M. Deary I. J. (2020). Physical frailty and decline in general and specific cognitive abilities: the Lothian birth cohort 1936. *J. Epidemiol. Community Health* 74 108–113. 10.1136/jech-2019-213280 31690586 PMC6993023

[B23] Gallucci M. Piovesan C. Di Battista M. E. (2018). Associations between the frailty index and brain atrophy: the Treviso dementia (TREDEM) registry. *J. Alzheimers Dis.* 62 1623–1634. 10.3233/jad-170938 29504533

[B24] Gobbens R. J. Luijkx K. G. Wijnen-Sponselee M. T. Schols J. M. (2010). In search of an integral conceptual definition of frailty: opinions of experts. *J. Am. Med. Dir. Assoc.* 11 338–343. 10.1016/j.jamda.2009.09.015 20511101

[B25] Gopalakrishna G. Mustafa R. A. Davenport C. Scholten R. J. Hyde C. Brozek J. (2014). Applying grading of recommendations assessment, development and evaluation (GRADE) to diagnostic tests was challenging but doable. *J. Clin. Epidemiol.* 67 760–768. 10.1016/j.jclinepi.2014.01.006 24725643

[B26] Grant R. L. (2014). Converting an odds ratio to a range of plausible relative risks for better communication of research findings. *BMJ* 348:f7450. 10.1136/bmj.f7450 24464277

[B27] Gray S. L. Anderson M. L. Hubbard R. A. LaCroix A. Crane P. K. McCormick W. (2013). Frailty and incident dementia. *J. Gerontol. A Biol. Sci. Med. Sci.* 68 1083–1090. 23419778 10.1093/gerona/glt013 PMC3738027

[B28] Higgins J. P. Thompson S. G. Deeks J. J. Altman D. G. (2003). Measuring inconsistency in meta-analyses. *BMJ* 327 557–560. 10.1136/bmj.327.7414.557 12958120 PMC192859

[B29] Huang C. H. Okada K. Matsushita E. Uno C. Satake S. Martins B. A. (2021). The association of social frailty with intrinsic capacity in community-dwelling older adults: a prospective cohort study. *BMC Geriatr.* 21:515. 10.1186/s12877-021-02466-6 34579661 PMC8475329

[B30] KelaiditiE. CesariM. CanevelliM. van KanG. A. OussetP. J. Gillette-GuyonnetS. (2013). Cognitive frailty: rational and definition from an (I.A.N.A./I.A.G.G.) international consensus group. *J. Nutr. Health Aging* 17 726–734. 10.1007/s12603-013-0367-2 24154642

[B31] Li M. Huang Y. Liu Z. Shen R. Chen H. Ma C. (2020). The association between frailty and incidence of dementia in Beijing: findings from 10/66 dementia research group population-based cohort study. *BMC Geriatr.* 20:138. 10.1186/s12877-020-01539-2 PMC7158148 32293307

[B32] Liu S. Kang L. Liu X. Zhao S. Wang X. Li J. (2021). Trajectory and correlation of intrinsic capacity and frailty in a Beijing elderly community. *Front. Med.* 8:751586. 10.3389/fmed.2021.751586 34957141 PMC8695757

[B33] Liu Z. Hsu F. C. Trombetti A. King A. C. Liu C. K. Manini T. M. (2018). Effect of 24-month physical activity on cognitive frailty and the role of inflammation: the LIFE randomized clinical trial. *BMC Med.* 16:185. 10.1186/s12916-018-1174-8 30352583 PMC6199791

[B34] Livingston G. Huntley J. Sommerlad A. Ames D. Ballard C. Banerjee S. (2020). Dementia prevention, intervention, and care: 2020 report of the lancet commission. *Lancet* 396 413–446. 10.1016/s0140-6736(20)30367-6 32738937 PMC7392084

[B35] Magnuson A. Lei L. Gilmore N. Kleckner A. S. Lin F. V. Ferguson R. (2019). Longitudinal relationship between frailty and cognition in patients 50 years and older with breast cancer. *J. Am. Geriatr. Soc.* 67 928–936. 10.1111/jgs.15934 31034595 PMC6490967

[B36] Moher D. Liberati A. Tetzlaff J. Altman D. G. (2010). Preferred reporting items for systematic reviews and meta-analyses: the PRISMA statement. *Int. J. Surg.* 8 336–341. 10.1016/j.ijsu.2010.02.007 20171303

[B37] Montero-Odasso M. M. Barnes B. Speechley M. Muir Hunter S. W. Doherty T. J. Duque G. (2016). Disentangling cognitive-frailty: results from the gait and brain study. *J. Gerontol. A Biol. Sci. Med. Sci.* 71 1476–1482. 10.1093/gerona/glw044 26984391

[B38] Morley J. E. Vellas B. van Kan G. A. Anker S. D. Bauer J. M. Bernabei R. (2013). Frailty consensus: a call to action. *J. Am. Med. Dir. Assoc.* 14 392–397. 10.1016/j.jamda.2013.03.022 23764209 PMC4084863

[B39] O’Caoimh R. Sezgin D. O’Donovan M. R. Molloy D. W. Clegg A. Rockwood K. (2021). Prevalence of frailty in 62 countries across the world: a systematic review and meta-analysis of population-level studies. *Age Ageing* 50 96–104. 10.1093/ageing/afaa219 33068107

[B40] Orrell M. Brayne C. (2015). Dementia prevention: call to action. *Lancet* 386:1625. 10.1016/s0140-6736(15)00528-0 26595627

[B41] Panegyres P. K. (2004). The contribution of the study of neurodegenerative disorders to the understanding of human memory. *QJM* 97 555–567. 10.1093/qjmed/hch096 15317924

[B42] Panza F. Lozupone M. Logroscino G. (2019). Understanding frailty to predict and prevent dementia. *Lancet Neurol.* 18 133–134. 10.1016/s1474-4422(18)30446-0 30663601

[B43] Panza F. Solfrizzi V. Barulli M. R. Santamato A. Seripa D. Pilotto A. (2015). Cognitive frailty: a systematic review of epidemiological and neurobiological evidence of an age-related clinical condition. *Rejuvenation Res.* 18 389–412. 10.1089/rej.2014.1637 25808052

[B44] Paolillo E. W. Sun-Suslow N. Pasipanodya E. C. Morgan E. E. Ellis R. J. Jeste D. V. (2020). Pre-frailty predicts cognitive decline at 2-year follow-up in persons living with HIV. *J. NeuroVirol.* 26 168–180. 10.1007/s13365-019-00814-2 31858484 PMC7391910

[B45] Qiu Y. Li G. Wang X. Zheng L. Wang C. Wang C. (2022). Prevalence of cognitive frailty among community-dwelling older adults: a systematic review and meta-analysis. *Int. J. Nurs. Stud.* 125:104112. 10.1016/j.ijnurstu.2021.104112 34758429

[B46] Riley R. D. Higgins J. P. Deeks J. J. (2011). Interpretation of random effects meta-analyses. *BMJ* 342:d549. 10.1136/bmj.d549 21310794

[B47] Robertson D. A. Savva G. M. Kenny R. A. (2013). Frailty and cognitive impairment–a review of the evidence and causal mechanisms. *Ageing Res. Rev.* 12 840–851. 10.1016/j.arr.2013.06.004 23831959

[B48] Rogers N. T. Steptoe A. Cadar D. (2017). Frailty is an independent predictor of incident dementia: evidence from the english longitudinal study of ageing. *Sci. Rep.* 7:15746. 10.1038/s41598-017-16104-y PMC5691042 29146957

[B49] Ruan Q. Yu Z. Chen M. Bao Z. Li J. He W. (2015). Cognitive frailty, a novel target for the prevention of elderly dependency. *Ageing Res. Rev.* 20 1–10. 10.1016/j.arr.2014.12.004 25555677

[B50] Shimada H. Doi T. Lee S. Makizako H. Chen L. K. Arai H. (2018a). Cognitive frailty predicts incident dementia among community-dwelling older people. *J. Clin. Med.* 7:250. 10.3390/jcm7090250 30200236 PMC6162851

[B51] ShimadaH. MakizakoH. TsutsumimotoK. DoiT. LeeS. SuzukiT. (2018b). Cognitive frailty and incidence of dementia in older persons. *J. Prev. Alzheimers Dis.* 5 42–48. 10.14283/jpad.2017.29 29405232

[B52] Solfrizzi V. Scafato E. Frisardi V. Seripa D. Logroscino G. Maggi S. (2013). Frailty syndrome and the risk of vascular dementia: the Italian longitudinal study on aging. *Alzheimers Dement.* 9 113–122. 10.1016/j.jalz.2011.09.223 23245560

[B53] Solfrizzi V. Scafato E. Lozupone M. Seripa D. Giannini M. Sardone R. (2017a). Additive role of a potentially reversible cognitive frailty model and inflammatory state on the risk of disability: the Italian longitudinal study on aging. *Am. J. Geriatr. Psychiatry* 25 1236–1248. 10.1016/j.jagp.2017.05.018 28689645

[B54] Solfrizzi V. Scafato E. Lozupone M. Seripa D. Schilardi A. Custodero C. (2019). Biopsychosocial frailty and the risk of incident dementia: the Italian longitudinal study on aging. *Alzheimers Dement.* 15 1019–1028. 10.1016/j.jalz.2019.04.013 31278052

[B55] Solfrizzi V. Scafato E. Seripa D. Lozupone M. Imbimbo B. P. D’Amato A. (2017b). Reversible cognitive frailty, dementia, and all-cause mortality. The Italian longitudinal study on aging. *J. Am. Med. Dir. Assoc.* 18 89.e1–89.e8. 10.1016/j.jamda.2016.10.012 28012505

[B56] Stroup D. F. Berlin J. A. Morton S. C. Olkin I. Williamson G. D. Rennie D. (2000). Meta-analysis of observational studies in epidemiology: a proposal for reporting. Meta-analysis of observational studies in epidemiology (MOOSE) group. *Jama* 283 2008–2012. 10.1001/jama.283.15.2008 10789670

[B57] Sugimoto T. Ono R. Kimura A. Saji N. Niida S. Sakai T. (2020). Impact of cognitive frailty on activities of daily living, cognitive function, and conversion to dementia among memory clinic patients with mild cognitive impairment. *J. Alzheimers Dis.* 76 895–903. 10.3233/jad-191135 32568192

[B58] Tarazona-Santabalbina F. J. Gómez-Cabrera M. C. Pérez-Ros P. Martínez-Arnau F. M. Cabo H. Tsaparas K. (2016). A multicomponent exercise intervention that reverses frailty and improves cognition, emotion, and social networking in the community-dwelling frail elderly: a randomized clinical trial. *J. Am. Med. Dir. Assoc.* 17 426–433. 10.1016/j.jamda.2016.01.019 26947059

[B59] Teo N. Yeo P. S. Gao Q. Nyunt M. S. Z. Foo J. J. Wee S. L. (2019). A bio-psycho-social approach for frailty amongst Singaporean Chinese community-dwelling older adults – evidence from the Singapore longitudinal aging study. *BMC Geriatr.* 19:350. 10.1186/s12877-019-1367-9 31830924 PMC6909571

[B60] Thibeau S. McDermott K. McFall G. P. Rockwood K. Dixon R. A. (2019). Frailty effects on non-demented cognitive trajectories are moderated by sex and Alzheimer’s genetic risk. *Alzheimers Res. Ther.* 11:55. 10.1186/s13195-019-0509-9 PMC6587247 31221191

[B61] Trebbastoni A. Canevelli M. D’Antonio F. Imbriano L. Podda L. Rendace L. (2017). The impact of frailty on the risk of conversion from mild cognitive impairment to Alzheimer’s disease: evidences from a 5-year observational study. *Front. Med.* 4:178. 10.3389/fmed.2017.00178 29109949 PMC5660054

[B62] Tsutsumimoto K. Doi T. Makizako H. Hotta R. Nakakubo S. Makino K. (2017). Association of social frailty with both cognitive and physical deficits among older people. *J. Am. Med. Dir. Assoc.* 18 603–607. 10.1016/j.jamda.2017.02.004 28411094

[B63] Tsutsumimoto K. Doi T. Nakakubo S. Kim M. Kurita S. Ishii H. (2019). Impact of social frailty on Alzheimer’s disease onset: a 53-month longitudinal cohort study. *J. Alzheimers Dis.* 70 585–593. 10.3233/JAD-181178 31256123

[B64] Veronese N. Custodero C. Cella A. Demurtas J. Zora S. Maggi S. (2021). Prevalence of multidimensional frailty and pre-frailty in older people in different settings: a systematic review and meta-analysis. *Ageing Res. Rev.* 72:101498. 10.1016/j.arr.2021.101498 34700009 PMC12149324

[B65] Wallace L. Hunter S. Theou O. Fleming J. Rockwood K. Brayne C. (2021). Frailty and neuropathology in relation to dementia status: the Cambridge City over-75s Cohort study. *Int. Psychogeriatr.* 33 1035–1043. 10.1017/s1041610220003932 33586645

[B66] Wallace L. M. K. Theou O. Godin J. Andrew M. K. Bennett D. A. Rockwood K. (2019). Investigation of frailty as a moderator of the relationship between neuropathology and dementia in Alzheimer’s disease: a cross-sectional analysis of data from the rush memory and aging project. *Lancet Neurol.* 18 177–184. 10.1016/s1474-4422(18)30371-5 30663607 PMC11062500

[B67] Ward D. D. Ranson J. M. Wallace L. M. K. Llewellyn D. J. Rockwood K. (2021). Frailty, lifestyle, genetics and dementia risk. *J. Neurol. Neurosurg. Psychiatry* 93, 343–350. 10.1136/jnnp-2021-327396 34933996 PMC8921595

[B68] WHO (2022). *Dementia.* Available online at: https://www.who.int/health-topics/dementia#tab=tab_1 (accessed January 5, 2021).

[B69] Williams A. M. Krull K. R. Howell C. R. Banerjee P. Brinkman T. M. Kaste S. C. (2021). Physiologic frailty and neurocognitive decline among young-adult childhood cancer survivors: a prospective study from the ST Jude lifetime cohort. *J. Clin. Oncol.* 39 3485–3495. 10.1200/jco.21.00194 34283634 PMC8547937

[B70] Winblad B. Amouyel P. Andrieu S. Ballard C. Brayne C. Brodaty H. (2016). Defeating Alzheimer’s disease and other dementias: a priority for European science and society. *Lancet Neurol.* 15 455–532. 10.1016/s1474-4422(16)00062-4 26987701

[B71] Yu J. T. Xu W. Tan C. C. Andrieu S. Suckling J. Evangelou E. (2020). Evidence-based prevention of Alzheimer’s disease: systematic review and meta-analysis of 243 observational prospective studies and 153 randomised controlled trials. *J. Neurol. Neurosurg. Psychiatry* 91 1201–1209. 10.1136/jnnp-2019-321913 32690803 PMC7569385

