# Supplementary material for: Multi-Concept Frailty Predicts the Late-Life Occurrence of Cognitive Decline or Dementia: An Updated Systematic Review and Meta-Analysis of Longitudinal Studies
Source: Front Aging Neurosci. 2022 May 11;14:855553. doi: 10.3389/fnagi.2022.855553 (PMC9131093; doi:10.3389/fnagi.2022.855553)
Supplement: Supplementary file 1 [file Data_Sheet_1.PDF]

Appendix

Appendix 1 Newcastle-Ottawa Quality Assessment Scale- Cohort Studies (involving version)[&] 1
Appendix 2 Approaches used to assess the credibility of meta-analysis. 4
Appendix 3 Supplementary tables and figures. 5
Table S1 Supplementary characteristics of included studies. 5
Table S2 The corresponding assessment scale of diverse frailty. 8
Table S3 Relationships between frailty and performance in specific cognitive domains over time. 9
Table S4 Credibility of meta-analyses results. 11
Figure S1 Subgroup analyses for the association between physical frailty and dementia or cognitive decline. 13

Appendix 1 Newcastle-Ottawa Quality Assessment Scale- Cohort Studies (involving version)[&]

Risk of bias	Questions	Scores	Standards
**Selection (generalisability, assessment bias and potential reverse causality)**	Q1. representativeness of the exposed cohort	☆	a) randomly selected or<br>b) database covering very large population or<br>c) participation rate (PR) is ≥ 90% or<br>d) reported there is no difference in important characteristics between those who agreed to participate and those who did not
		0.5☆	PR varies from 70% to 90% with no reporting of significant difference in important characteristics between those who agreed to participate and those who did not
		0	Selected group of users e.g. nurses, volunteers or no description
	Q2. selection of the non-exposed cohort	☆	Drawn from the same community as the exposed cohort
		0	Drawn from a different source or no description of the derivation of the non-exposed cohort
	Q3. ascertainment of exposure	☆	Questionnaire or interview based on self-report to series questions or database
		0.5☆	Self-report to simple question with potential recall bias
		0	No description
	Q4. demonstration that outcome of interest was not present at baseline	☆	Cognitively intact for outcome as dementia or MCI; Free of dementia for population with MCI at baseline
		0.5☆	Free of dementia (cognitively intact & cognitive impairment no dementia (CIND)) for outcome of dementia
		0	No description
**Confounding bias**	Q5. comparability of cohorts on the basis of the design or analysis	☆☆	Except for age, sex, and education, the analysis still controls for at least another two domains of AD risk factors, including *APOE4*, pre-existing disease, lifestyle, medical exposure, biochemical exposure, occupation, diet, etc.
		☆	Controls for age, sex and education
		0	No description
**Outcome (assessment bias and attrition**	Q6. assessment of outcome	☆	Independent or blind assessment
		0.5☆	Record linkage (e.g. identified through ICD codes on database records or claim data)
		0	Self-report or no description

**bias)**	Q7. follow-up long enough for outcomes to occur?#	☆	The average or max duration reached the lower 95% CI.
		0	The average or max duration did not reach the lower 95% CI.
	Q8. adequacy of follow up of cohorts*	☆	Attrition rate ≤ 5%
		0.5☆	5% ≤ Attrition rate ≤ 20%
		0	Attrition rate > 20% and no description of those lost or no description

& A study can be awarded a maximum of one star for each numbered item within the Selection and Outcome categories. A maximum of two stars can be given for Comparability. In the involving version, an assignment of a half point (0.5) is permitted.

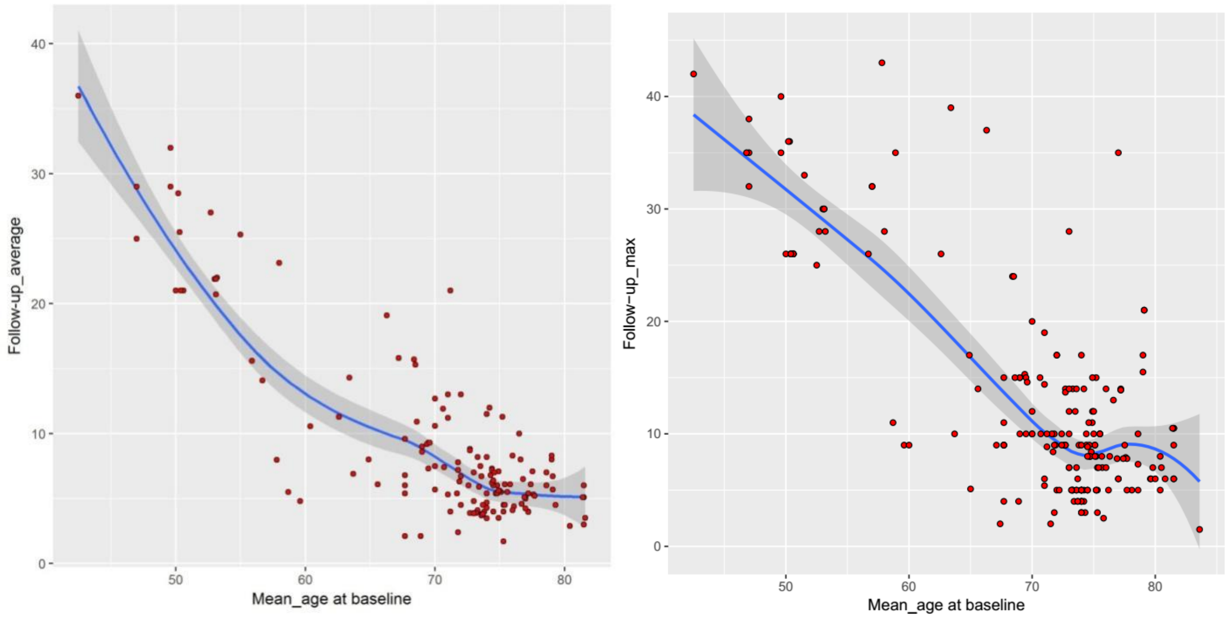

#Figure: It is obviously absurd to define a common period for population with diverse age range at baseline. A presumable negative correlation was reasonably supposed to exist between so-called adequate follow-up period and average age of population at baseline. Here, we will draw the nonlinear regression line with its 95% confidence interval (CI) for the association between the mean/max follow-up duration and mean age of population at baseline for AD cohorts (unpublished data). We will predefine that the follow-up is adequate if the average or max duration reach the lower 95% CI.

*It has been indicated that a rate of loss < 5% probably leads to little bias, whereas a rate of loss that is greater than 20% potentially poses serious threats to validity (2).

Reference

1. Stang A. Critical evaluation of the Newcastle-Ottawa scale for the assessment of the quality of nonrandomized studies in meta-analyses. Eur J Epidemiol (2010) 25(9):603-5. Epub 2010/07/24. doi: 10.1007/s10654-010-9491-z. PubMed PMID: 20652370.
2. Sackett DL, Richardson WS, Rosenberg W, Haynes RB (1997). Evidence-based medicine: how to practice and teach EBM. NewYork: Churchill Livingstone.

Appendix 2 Approaches used to assess the credibility of meta-analysis.

1. Inconsistency

We estimated the inconsistency according to the value of I^2, which ranged from 0% to 100%. 0% represented no inconsistency.

2. Imprecision

Calculate the RR & 95% confidence interval (CI) and 95% prediction interval (PI):

- The CI in a random-effects model contains highly probable values for the summary (mean)effect but gives no information on the range of true effects that are likely to be seen in other settings which convey what range of intervention effects are likely to be seen in otherindividuals.
- A 95% PI estimates where the true effects are to be expected for 95% of similar (exchangeable) studies that might be conducted in the future. In the absence of between-study heterogeneity, the prediction interval coincides with the respective CI. However, in case of heterogeneity, a prediction interval covers a wider range than a CI.

95%CI	95%PI	Rating
Not containing RR=1	Not containing RR=1	3
Containing RR=1 & not containing RR=0.75 or 1.25	Containing RR=1 & not containing RR=0.75 or 1.25	3
Not containing RR=1	Containing RR=1	2
Containing RR=1 & not containing RR=0.75 or 1.25	Containing RR=1 & RR=0.75 or 1.25	2
Containing RR=1 & 0.75 or 1.25	Containing RR = 1 & 0.75 or 1.25 or PI not available	1

Note: RR=0.75 or 1.25 is representative of the rough cutoff of evident benefits or harm.

3. Risk of bias:

We used the score of weighted quality score (WQS) to assess the risk of bias, the calculation formula is as follows:

WQS=QS (study 1) × weight% (study 1) + QS (study 2) × weight% (study 2) + ……QS (study n) × weight% (study n)

Note: “QS” means NOS score (total score= 9); “Weight” means weight value in the random model.

4. Publication bias

The P-value of the Egger test was used to assess the publication bias, which ranged from 0 to 1. The publication bias improved with the decrease of P-value.

5. Indirectness

To assess whether frailty increases the risk of dementia, we assigned various outcomes as following scores: AD=3, dementia=3, dementia or cognitive decline=2, cognitive decline=1.

Reference

1. Gopalakrishna G, Mustafa RA, Davenport C, Scholten RJ, Hyde C, Brozek J, et al. Applying Grading of Recommendations Assessment, Development and Evaluation (GRADE) to diagnostic tests was challenging but doable. J Clin Epidemiol (2014) 67(7):760-8. Epub 2014/04/15. doi: 10.1016/j.jclinepi.2014.01.006. PubMed PMID: 24725643.
2. Guyatt GH, Oxman AD, Vist G, Kunz R, Brozek J, Alonso-Coello P, et al. GRADE guidelines: 4. Rating the quality of evidence--study limitations (risk of bias). J Clin Epidemiol (2011) 64(4):407-15. Epub 2011/01/21. doi: 10.1016/j.jclinepi.2010.07.017. PubMed PMID: 21247734.
3. Guyatt GH, Oxman AD, Kunz R, Brozek J, Alonso-Coello P, Rind D, et al. GRADE guidelines 6. Rating the quality of evidence--imprecision. J Clin Epidemiol (2011) 64(12):1283-93. Epub 2011/08/16. doi: 10.1016/j.jclinepi.2011.01.012. PubMed PMID: 21839614.
4. Guyatt GH, Oxman AD, Kunz R, Woodcock J, Brozek J, Helfand M, et al. GRADE guidelines: 8. Rating the quality of evidence--indirectness. J Clin Epidemiol (2011) 64(12):1303-10. Epub 2011/08/02. doi: 10.1016/j.jclinepi.2011.04.014. PubMed PMID: 21802903.

Appendix 3 Supplementary tables and figures.

Table S1 Supplementary characteristics of included studies.

First author, year	Study design	Sample source	Participation rate	Attrition rate	Effect measure	Diagnostic methods of outcome	Adjusted confounders
Buchman, 2007	PRO	Community	51%	18%	HR	Single clinician	Age, gender, and education.
Avila-Funes, 2009	PRO	Others	37%*	17.6%*	HR	Consensus	Age, sex, education level, income, smoking status, drinker status, number of chronic diseases, self-reported health, Center for Epidemiologic Studies Depression Scale score, mobility, APOE-e4 allele, instrumental activity of daily living, and activity of daily living disability at baseline.
Boyle, 2010	PRO	Others	51%	na	HR	Single clinician	Age, sex, and education.
Avila-Funes, 2012	PRO	Others	37%	10%	HR	Consensus	Sex, educational level, APOE e4, hypertension, diabetes mellitus, hypercholesterolemia, smoking status, prevalent myocardial infarction, and activity of daily living disability.
Gray, 2013	PRO	Others	48%	34%	HR	Consensus	Age at baseline, sex, education, race, body mass index, depressive symptoms, antidepressant use, self-reported health, hypertension, diabetes, myocardial infarction, congestive heart failure, and smoking status and baseline Cognitive Abilities Screening Instrument score.
Solfrizzi, 2013	PRO	Others	47%	0%	HR	Single clinician	Age categories, sex, education, pack-years, instrumental activities of daily living score, MMSE score at baseline, Charlson comorbidity index score, and serum albumin levels.
Montero-Odasso, 2016	PRO	Community	na	na	HR	Single clinician	Age, sex, years of education, number of comorbidities.
Feng, 2017	PRO	Community	72.4%*	33.59%*	OR	Consensus	Age, gender, education, diabetes, heart failure, atrial fibrillation, current smoking, alcohol drinking, APOE-e4 carrying status, and depressive symptoms.
Rogers, 2017	PRO	Community	94%	19%	HR	Self-report	Sex, age, wealth, educational qualifications, living alone, alcohol intake, physical inactivity, and smoking status.
Solfrizzi, 2017a	PRO	Others	43%	na	IRR	Single	Age, sex, education, pack-years cigarettes, Charlson Comorbidity Index, and

						clinician	apolipoprotein B/apolipoprotein A-I.
Solfrizzi, 2017b	PRO	Others	39%	na	HR	Single clinician	Age categories, sex, education, pack-years, GDS-30 score, IADL score, MMSE score at baseline, Charlson comorbidity index score, and serum albumin levels.
Trebbastoni, 2017	RETRO	Organization	—	—	HR	na	Age, sex, and MMSE.
Chen, 2018	PRO	Community	54%	31%	OR	—	Age, gender, years of education heart disease, pulmonary disease, hypertension, hyperlipidemia, diabetes mellitus, osteoarthritis, stroke, and depression, medications, living alone, current smoking, exercise habit, engaging in paid work, subjective memory complaints, GDS score, and certified long-term care insurance and functional limitations.
Shimada, 2018a	PRO	Community	47%	4%	HR	Single clinician	Age, sex, education level, heart disease, pulmonary disease, hypertension, hyperlipidemia, diabetes, osteoarthritis, stroke, depression, medications, living alone, current smoking, exercise habit, engaging in paid work, subjective memory complaints, GDS score, Japanese-certified public long-term care insurance system, functional limitations.
Shimada, 2018b	PRO	Community	47%	2%	HR	Single clinician	Age, sex, educational level, GDS score, smoking, heart disease, pulmonary disease, hypertension, hyperlipidemia, and diabetes.
Bunce, 2019	PRO	Others	69%	61%	—	—	No.
Magnuson, 2019	PRO	Organization	na	11%	—	—	Age, race, marital status, education, performance status, baseline anxiety, baseline depression, and baseline frailty score.
Solfrizzi, 2019	PRO	Others	40%	na	HR	Single clinician	Age categories, gender, education, pack-years, instrumental activities of daily living score, MMSE score at baseline, Charlson Comorbidity Index score, serum albumin levels, and apolipoprotein B to apolipoprotein A-1 ratio.
Thibeau, 2019	PRO	Community	91%	10-23%	—	—	No.
Tsutsumimoto, 2019	PRO	Community	36%	10%	HR	Single clinician	Sociodemographic factors (age, sex, education), medical conditions (BMI, polypharmacy, hypertension, diabetes, hyperlipidemia), lifestyle factors (physical activity, current drinking habit, and current smoking habit), physical and neuropsychological functioning (muscle weakness, cognitive decline, and depressive symptoms).
Gale, 2020	PRO	Community	29%	>30%	—	—	Age, sex, education, depressive symptoms, smoking, and number of chronic illnesses.
Li, 2020	PRO	Community	80%	19.1%	HR	na	Age, gender, education level, physical multimorbidity, walking distance, smoking status,

							and alcohol use.
Paolillo, 2020	PRO	Organization	na	na	—	—	No.
Sugimoto, 2020	RETRO	Organization	na	na	HR	na	Age, sex, education, living situation, smoking status, alcohol consumption, Barthel Index score, MMSE score, GDS-15 score, DBD score, and APOE status.
Williams, 2021	PRO	Organization	na	9%	—	—	No
Bai, 2021	PRO	Organization	69%	73%*	HR	Consensus	Age, gender, education, cognitive function score, and Tobacco use.
Chen, 2021	PRO	Community	na	14%	—	—	Age, sex, years of education, APOE e4 status, alcohol drinking, number of chronic diseases, body mass index, follow-up time, the other frailty dimension.
Liu, 2021	PRO	Community	na	19%	—	—	Age, polypharmacy, and Charlson Comorbidity Index.
Ward, 2021	PRO	Others	na	10%	HR	Single clinician	Age at baseline, sex, education level, socioeconomic status, the polygenic risk score, the number of alleles included in the polygenic.
Huang, 2021	PRO	Community	100%	37%	—	—	Age, sex, educational level, BMI, CCI score, and BAQ score.

* As the information of sample wasn't accessible, the total participation information was used as a proxy.

APOE, apolipoprotein E; BAQ, the Baecke Physical Activity Questionnaire. BMI, body mass index; CCI, the Charlson Comorbidity Index; DBD, Dementia Behavior Disturbance scale; GDS, Geriatric Depression Scale; HR, hazard ratio; IADL, instrumental activities of daily living; IRR, incidence rate ratios; MMSE, Mini-Mental State Examination; na, not applicable; OR, odds ratio; PRO, prospective study; RETRO, retrospective study.

Table S2 The corresponding assessment scale of diverse frailty.

Type of frailty	Assessment scales
Physical frailty/prefrailty	Frailty Phenotype scale;<br>Physical frailty index scale
Cognitive frailty/prefrailty	Frailty Phenotype scale + cognitive impairment;<br>Physical frailty index scale + cognitive impairment
Potentially reversible cognitive frailty	Frailty Phenotype scale + MCI
Reversible cognitive frailty	Frailty Phenotype scale + SCD
Social frailty	Assessment instruments based on the concept of social frailty for each study are not uniform.
Biopsychosocial frailty	Multidimensional frailty index scale

Table S3 Relationships between frailty and performance in specific cognitive domains over time.

First author, year	Study population	Type of frailty and frailty assessment	Measure of cognition	Results
Boyle, 2010	Community-dwelling older.	PF: composite measure of grip strength, timed walk, body composition and fatigue.	Global cognition, episodic memory, semantic memory, working memory, perceptual speed, and visuospatial abilities.	Frailty associated with more rapid decline in global cognition and all cognitive domains.
Bunce, 2019	Old people recruited from the electoral rolls.	PF: mPF	Global cognition, processing speed, verbal fluency, face and word recognition, episodic memory and simple and choice reaction time.	Frailty had no effect on any slopes of cognition.
Magnuson, 2019	Breast cancer patients receiving adjuvant/ neoadjuvant chemotherapy and control participants from clinics.	PF: a modified score based on mPF.	Global cognitive function, visual memory, sustained attention, verbal fluency, attention, verbal memory.	Longitudinal decline in global cognitive function, verbal fluency, attention and visual memory was associated with increased frailty score in patients compared with controls.
Thibeau, 2019	Community-dwelling older.	PF: FI	Memory, speed, and executive function.	Frailty levels predicted differential memory change slopes. Change in frailty predicted the rate of decline for speed and executive function.
Gale, 2020	Community-dwelling older.	PF: mPF	Global cognitive function, visuospatial ability, memory, processing speed and crystallised ability.	Physical frailty, but not prefrailty, was associated with greater decline in general cognitive ability and all cognitive domain.
Paolillo, 2020	PLWH and HIV- individuals from the Multi-Dimensional Successful Aging.	PF: mPF	Global cognitive function, verbal fluency, executive function processing speed, learning, delayed recall, working memory, and motor skills.	From baseline to two-year follow-up, among PLWH, prefrail individuals demonstrated consistent declines in global cognition, processing speed, and motor function. Among HIV- participants, prefrail individuals declined in global cognition and processing speed.

Williams, 2021	Young-adult childhood cancer survivors from the St Jude Lifetime Cohort.	PF: mPF	Global cognition, academics, attention, processing speed, memory, executive function.	The frail survivors showed more declines in short-term verbal memory, visual-motor processing speed, cognitive flexibility and verbal fluency than the non-frail survivors. Prefrail and frail survivors had a greater reduction in focused attention than non-frail survivors.
Chen, 2021	Older adults from hospital.	PF: mPF; Psychosocial frailty: integrating self-rated health, mood, social relationship and support.	Global cognition, memory, attention, executive function, verbal fluency.	Greater physical frailty was associated with poor global cognition, logical memory and executive function. Greater psychosocial frailty was associated with poor global cognition and attention.

FI, frailty index; HIV, human immunodeficiency virus; HIV-, HIV-uninfected; mFP, modified Frailty phenotype; PF, physical frailty; PLWH, people living with HIV.

Table S4 Credibility of meta-analyses results.

Type of frailty		Max*	Min&	Dementia or cognitive decline	Cognitive decline	Dementia	AD
Physical frailty	Number of publications	…	…	11	4	7	4
	Inconsistency	0%	100%	21.1%	40.2%	0.456%	51.3%
	Imprecision	3	1	3	2	3	1
	Risk of bias	9	0	7.308	6.656	7.757	7.563
	Publication bias	1	0	0.795	0.855	0.05	0.758
	Indirectness	3	1	2	1	3	3
Physical prefrailty	Number of publications	…	…	6	3	4	2
	Inconsistency	0%	100%	66%	62.4%	65.2%	85.7%
	Imprecision	3	1	1	1	1	…
	Risk of bias	9	0	7.3255	6.701	7.569	7.74
	Publication bias	1	0	0.095	0.42	0.319	…
	Indirectness	3	1	2	1	3	3
Cognitive frailty	Number of publications	…	…	6	1	6	1
	Inconsistency	0%	100%	80.7%	…	78.1%	…
	Imprecision	3	1	1	…	2	…
	Risk of bias	9	0	7.206	6.00	7.214	8.00
	Publication bias	1	0	0.942	…	0.461	…
	Indirectness	3	1	2	1	3	3
Cognitive prefrailty	Number of publications	…	…	3	1	3	0
	Inconsistency	0%	100%	77.1%	…	30.2%	…
	Imprecision	3	1	2	…	2	…
	Risk of bias	9	0	7.296	8.00	7.634	…
	Publication bias	1	0	0.599	…	0.278	…
	Indirectness	3	1	2	1	3	3
Biopsychosocial frailty	Number of publications	…	…	6	0	5	2
	Inconsistency	0%	100%	95.8%	…	95.7%	0.0%
	Imprecision	3	1	2	…	2	…
	Risk of bias	9	0	7.137	…	7.736	5.01
	Publication bias	1	0	0.585	…	0.612	…
	Indirectness	3	1	2	1	3	3

[*]Max is the value represented by the outermost point on the radar chart. [&]Min is the value represented by the point in the center of the radar chart.

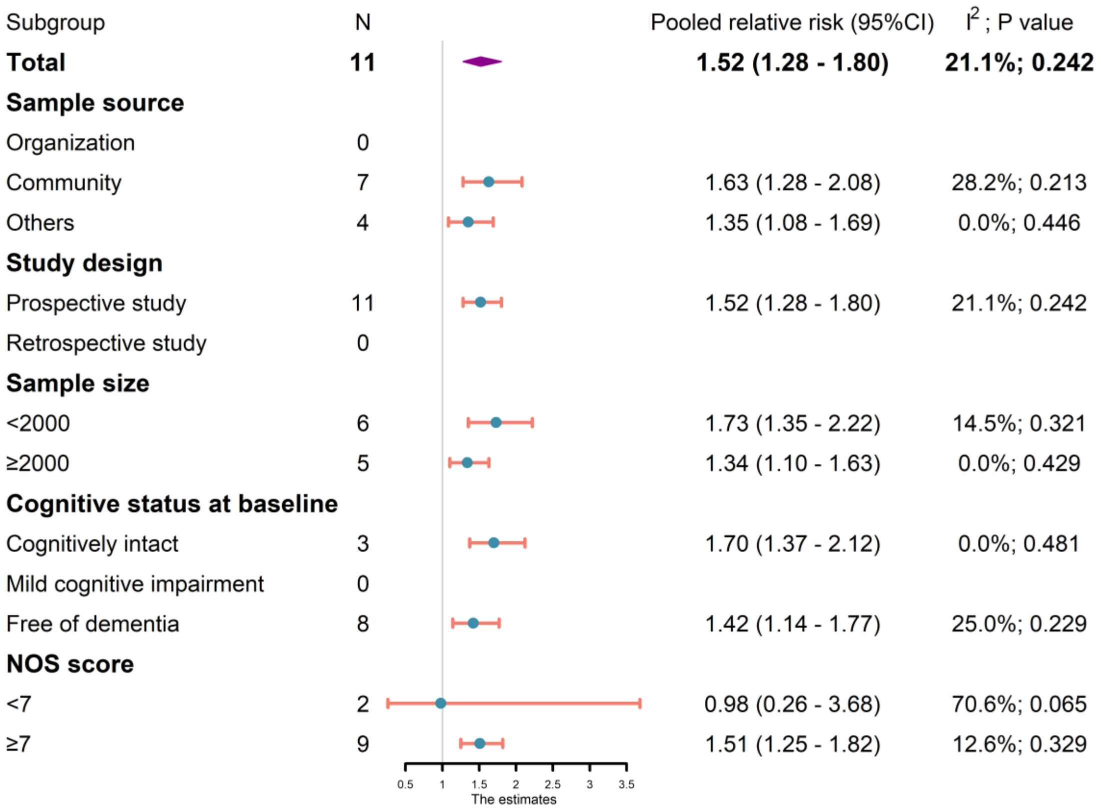

Figure S1. Subgroup analyses for the association between physical frailty and dementia or cognitive decline.

N, number of publications; CI, confidence interval.
